# An incarcerated internal hernia of the sigmoid colon through a defect in the broad ligament: A case report

**DOI:** 10.1016/j.ijscr.2021.106169

**Published:** 2021-07-07

**Authors:** Shinya Ohno, Wakana Chikaishi, Takuya Sugimoto, Shuji Komori, Masahiko Kawai

**Affiliations:** Department of Surgery, Gifu Prefectural General Medical Center, 4-6-1 Noisshiki, Gifu-City, Gifu-Prefecture 500-8717, Japan

**Keywords:** CRP, C-reactive protein, CT, computed tomography, Case report, Incarcerated hernia, Internal hernia, Broad ligament defect, Sigmoid colon

## Abstract

**Introduction and importance:**

Hernias through a defect of the broad ligament are rare, accounting for only 1.6–5% of internal herniations [1]. This report describes a rare case of sigmoid colon obstruction due to hernia through a defect of the broad ligament, which was diagnosed before surgery.

**Case presentation:**

A 78-year-old multiparous woman presented with lower abdominal pain and nausea. Contrast-enhanced multi-detector CT (MDCT) demonstrated a dilated sigmoid colon and edematous mesentery of the sigmoid colon in the left Douglas' fossa, the uterus was compressed dorsally to the right and the left ovary was compressed ventrally. We diagnosed an internal broad ligament defect hernia with incarceration of the sigmoid colon, and performed emergency laparotomy. The necrotic sigmoid colon was resected and anastomosis was performed by the double stapling technique. The postoperative course was uneventful.

**Clinical discussion:**

We consider the treatment of hernia of sigmoid colon through a broad ligament defect.

**Conclusion:**

We recognize that there is a possibility that, in addition to the small intestine, proximally located organs may be incarcerated. In the case of the colon, we should choose the treatment method carefully according to whether or not the colon is expected to be necrotic.

## Highlights

• Hernia of the sigmoid colon through a broad ligament defect is rare.
• The approach is based on whether or not the sigmoid colon is necrotic.
• We can evaluate whether it is necrotic or not and reposition by colonoscopy.
• Anatomically proximal organs may be incarcerated through such defects.

## 1 Introduction

Hernia through a defect of the broad ligament is rare, accounting for only 1.6–5% of internal herniations [1]. In most cases, the small intestine is the incarcerated organ. Cases of sigmoid colon incarceration are very rare. The condition is difficult to diagnose preoperatively because of nonspecific clinical symptoms [1]. We report a case of sigmoid colon obstruction due to herniation through a defect of the broad ligament, which we diagnosed preoperatively. Furthermore, we also discuss the treatment strategy for sigmoid colon obstruction through a broad ligament defect. This work is in line with the SCARE 2020 criteria [2].

## 2 Case presentation

**Fig. 1:**
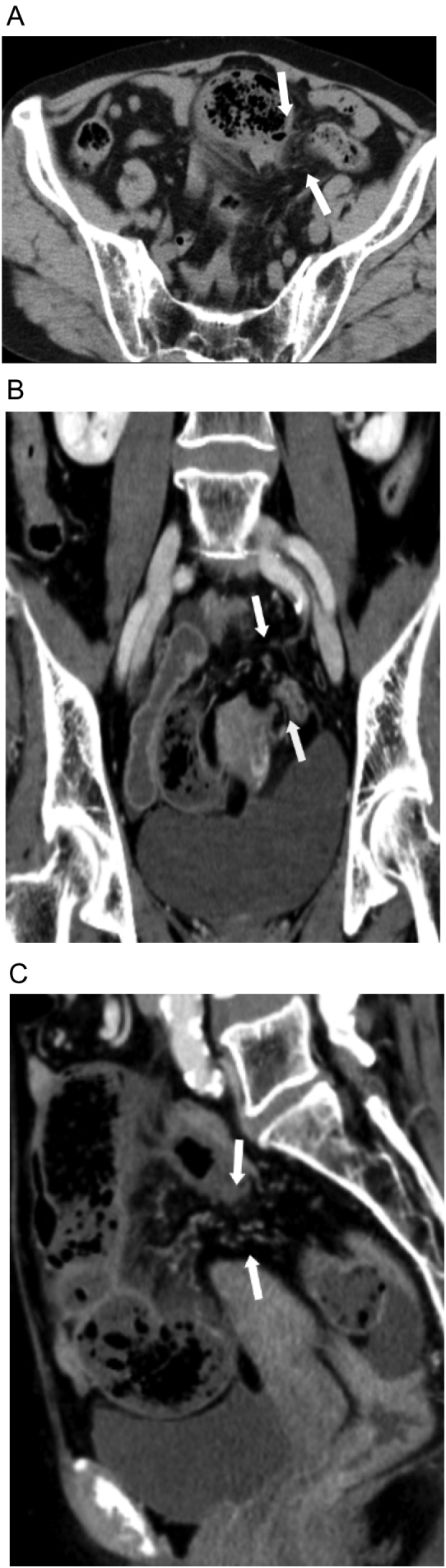
A computed tomography scan showing obstruction of sigmoid colon. Arrows show the defect of the broad ligament. A: Axial image: The uterus is compressed dorsally on the right, and the left ovary is compressed ventrally. B: Coronal image: Ascites collection is observed in Douglas' fossa. C: Sagittal image: Edematous mesentery of the sigmoid colon is observed in Douglas' fossa with ascites collection.

A 78-year-old multi-parous woman consulted her family doctor of lower abdominal pain and nausea, and was admitted to our hospital the following morning. There is no relevant past, surgical or family history, and she was not using any chronic medication. On physical examination, the abdomen was diffusely tender with no guarding or increased bowel sounds. Her vital signs were stable. Laboratory findings showed no definite abnormalities, with the exception of a slightly elevated white blood cell count (10,900/mm^3^) and CRP level (0.11 mg/dL). Contrast-enhanced MDCT showed on the one hand a dilated sigmoid colon and edematous mesentery of the sigmoid colon in Douglas' fossa with ascites collection, on the other hand there was no dilatation of the small intestine or air fluid level in the small intestine. The uterus was compressed to the right dorsally and the left ovary was compressed ventrally (Fig. 1A, B, C).

**Fig. 2:**
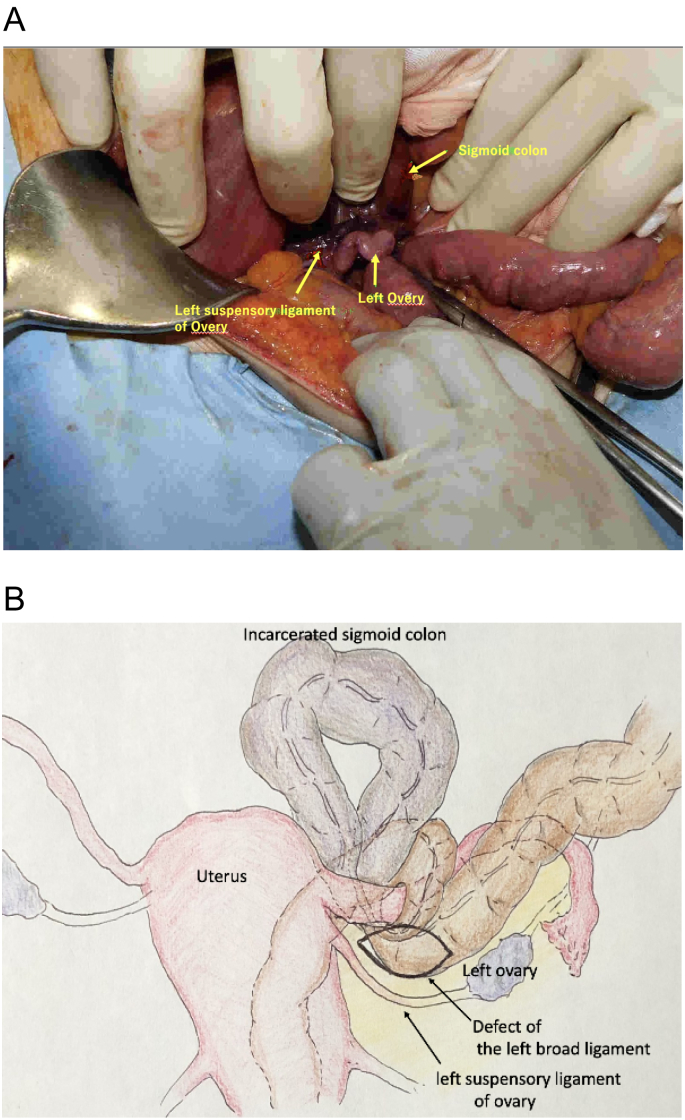
Intraoperative photograph and illustration. A: The sigmoid colon is incarcerated and necrotic. B: The sigmoid colon is herniating through the left broad ligament defect in a ventral to dorsal direction.

From these findings, we diagnosed an internal broad ligament defect hernia with incarceration of the sigmoid colon and performed emergency laparotomy without the transnasal drainage tube. A 15-cm loop of sigmoid colon had incarcerated by the left suspensory ligament of the ovary through a 3-cm defect of the left broad ligament in a ventral to dorsal direction and the sigmoid colon had become necrotic. The left suspensory ligament of the ovary and the left ovarian artery and vein were dissected and the left ovary was excised (Fig. 2A, B). The defect was released, largely to avoid recurrence of internal herniation. The segment of necrotic colon was resected and anastomosis was performed by the double stapling technique. The pathological diagnosis had no malignant findings. The postoperative course was uneventful.

## 3 Discussion

The defect is classified into 2 types: the fenestra type in which the anterior and posterior layer of the broad ligament are penetrated; and the pouch type, in which either the anterior or posterior leaf is penetrated and a blind pouch is formed [3]. Most defects are classified as the fenestra type as our patient. The main causes of defects of the broad ligament are congenital defects, external factors associated with pregnancy, delivery, surgery and hard labor, decreased elasticity due to aging, and inflammatory pelvic disease [3], [4], [5].

CT is useful for the diagnosis of hernias through broad ligament defects [4], [6]. The characteristic CT findings are reported to be as follows: bowel loop in Douglas' fossa, deviation of the pelvic organs by the dilated bowel, convergence of the bowels or congested mesentery close to the broad ligament and extension and deviation of the broad ligament [7], [8], [9].

In most cases involving incarcerated hernia through a broad ligament defect, the incarcerated organ is the small intestine, incarceration of the sigmoid colon is rare. Kawai et al. [10] reported that the colon is incarcerated less frequently than the small intestine because the wall of the colon is thick in comparison to the small intestine and has a low mobility due to fixation to the retroperitoneum and short mesentery; however, in the case of weak fixation to retroperitoneum of the sigmoid colon and when the sigmoid colon is long there is a possibility of its incarceration through a broad ligament defect, especially through the left side.

In recent years, the number of reports describing the laparoscopic treatment of hernia through broad ligament defects has increased [8]. Laparoscopic treatment has been reported to be associated with the following advantages in patients with this condition: 1) minimal invasion, 2) cosmetic superiority, 3) less pain, 4) useful for the diagnosis of ileus and the possibility of performing laparoscopic treatment, and 5) a minimal surgical wound if conversion to laparotomy is required [11], [12], [13]. However, we must secure sufficient working space for laparoscopy [14]. When poor visibility is expected due to the dilated bowel, the pressure of the bowel must be reduced by placement of a drainage tube. If the sigmoid colon is incarcerated but not expected to be necrotic, we can try to reposition of sigmoid colon with a digital examination and the performance of colonoscopy [10]. In colonoscopy, we can evaluate whether or not the colon is necrotic. However, we need to perform careful repositioning in order to avoid excessive loading of the bowels. In a non-necrotic and repositioned case, we can perform laparoscopy electively. To prevent recurrent bowel obstruction, the defect should be closed using a clip or suture [15], or the broad ligament should be completely divided [11].

In this case, instead of a laparoscopic approach, we chose to perform emergency laparotomy without the above-described preoperative procedure because we suspected necrosis of the sigmoid colon and visibility is expected to be poor due to dilatation of the sigmoid colon. In cases of hernia of the sigmoid colon, which is different from the small intestine, we should choose the treatment method carefully, it is difficult to maintain sufficient working space in the presence of the dilated bowels because mobility of the colon is limited in comparison to the small intestine and because preoperative transnasal drainage of the intestine has often no immediate effectivity or effort for the colon. Furthermore, when the bowel is necrotic, the risk of perforation and intraperitoneal contamination is higher.

## 4 Conclusion

To facilitate an early diagnosis, hernia through a defect of the broad ligament should be added to the list of differential diagnoses of female acute abdomen and it should be considered that in addition to the small intestine, proximally located organs may be incarcerated. In the case of the incarceration of the colon, we should choose the treatment method carefully according to whether or not the colon is expected to be necrotic.

## Sources of funding

This research did not receive any specific grant from funding agencies in the public, commercial, or not-for-profit sectors.

## Ethical approval

This report was reviewed and approved by the Institutional Review Board of Gifu Prefectural General Medical Center.

## Consent

Informed consent was obtained from the patient for publication of this case report.

## Author contributions

Shinya Ohno: Data Acquisition, Data Interpret and writing of the manuscript.

Wakana Chikaishi & Takuya Sugimoto: management of case.

Shuji Komori: Supervision, review and editing.

Masahiko Kawai: Supervision, review, editing, and final approval of the version to be submitted.

## Research registration

Not applicable.

## Guarantor

The guarantor is Shinya Ohno.

## Provenance and peer review

Not commissioned, externally peer-reviewed.

## Declaration of competing interest

The authors declare no conflicts of interest.
